# Enhanced Metabolic Effects of Fish Oil When Combined with Vitamin D in Diet-Induced Obese Male Mice

**DOI:** 10.3390/biom14040474

**Published:** 2024-04-12

**Authors:** Latha Ramalingam, Brennan Mabry, Kalhara R. Menikdiwela, Hanna Moussa, Naima Moustaid-Moussa

**Affiliations:** 1 Nutrigenomics, Inflammation and Obesity Research Laboratory, Department of Nutritional Sciences, Texas Tech University (TTU), Lubbock, TX 79409, USAkm1612@sebs.rutgers.edu (K.R.M.); 2 Obesity Research Institute, Office of Research & Innovation, Texas Tech University (TTU), Lubbock, TX 79409, USA; 3 Department of Physics & Astronomy, College of Arts & Sciences, Texas Tech University (TTU), Lubbock, TX 79409, USA

**Keywords:** fish oil, polyunsaturated fatty acids, vitamin D, obesity, mice studies

## Abstract

Vitamin D (vit D) and fish oil (FO) both offer unique health benefits, however, their combined effects have not been evaluated in obesity and nonalcoholic fatty liver disease (NAFLD). Hence, we hypothesized that vit D and FO supplementation would have additive effects in reducing obesity-associated inflammation and NAFLD. Male C57BL6 mice were split into four groups and fed a high fat (HF) diet supplemented with a low (HF; +200 IU vit D) or high dose of vitamin D (HF + D; +1000 IU vit D); combination of vit D and FO (HF-FO; +1000 IU vit D); or only FO (HF-FO; +200 IU vit D) for 12 weeks. We measured body weight, food intake, glucose tolerance, and harvested epididymal fat pad and liver for gene expression analyses. Adiposity was reduced in groups supplemented with both FO and vit D. Glucose clearance was higher in FO-supplemented groups compared to mice fed HF. In adipose tissue, markers of fatty acid synthesis and oxidation were comparable in groups that received vit D and FO individually in comparison to HF. However, the vit D and FO group had significantly lower fatty acid synthesis and higher oxidation compared to the other groups. Vit D and FO also significantly improved fatty acid oxidation, despite similar fatty acid synthesis among the four groups in liver. Even though we did not find additive effects of vit D and FO, our data provide evidence that FO reduces markers of obesity in the presence of adequate levels of vit D.

## 1. Introduction

Obesity is a complex disease and a significant health concern, afflicting more than 42% of the US adult population [1]. Obesity has also become a global health concern, affecting 16% of the world’s population [2]. Obesity is defined by excessive fat accumulation in adipose tissue, which increases the risk for other metabolic diseases, including Type 2 diabetes, cardiovascular diseases, and cancers [3]. Impairments in adipose tissue lead to excess secretion of adipose-related cytokines (adipokines) and pro-inflammatory cytokines, causing obesity-associated inflammation. Further, excess fatty acids from the adipose tissue causes ectopic fat accumulation in peripheral tissues, including the liver, contributing to non-alcoholic fatty liver disease (NAFLD) [4].

Both obesity and NAFLD are linked with high dietary intake of energy-dense and nutrient-poor sources of saturated and trans fats, as well as low intake of nutrient-dense sources of fiber and micronutrients (i.e., fruits and vegetables) [5,6,7]. Patients with obesity and NAFLD have micronutrient deficiencies, including insufficient vitamin D (vit D) levels [3,8,9,10]. Maintaining the recommended amount of vit D is critical, as it has multiple roles in metabolic health, immunity, and cardioprotective and neuroprotective effects [11,12,13]. Vit D’s role in calcium homeostasis and bone health is well established [14,15]. With regard to obesity, in prospective human studies, lower serum 25-hydroxy vitamin D (25D) levels were associated with higher incidence of obesity, NAFLD, and higher waist circumference [3,16]. Further, animal studies have shown that vit D supplementation to high-fat-fed rodents reduced their weight gain [17]. This may be in part due to increased fatty acid oxidation and reduced inflammation in adipose tissue [17,18]. Additionally, insufficient vit D levels caused more weight gain and adiposity [19], highlighting the importance of vit D in weight regulation.

Other potential bioactives known for their cardiometabolic protective properties are the omega-3 polyunsaturated fatty acids (n-3 PUFAs), namely eicosapentaenoic acid (EPA; 20:5*n*−3) and docosahexaenoic acid (DHA; 22:6*n*−3), that are found in oily fish or in dietary supplement forms [20]. The standard American diet is rich in n-6 compared to n-3 PUFAs, and studies show that omega 3 index (n-3 to n-6 ratio) is lower in patients with obesity [21]. Further, intake of n-3 PUFAs is known to reduce obesity in part through its hypertriglyceridemia and anti-inflammatory properties [22,23]. Preclinical studies have demonstrated that n-3 PUFAs reduce weight gain partly by reducing fatty acid synthesis and reducing inflammation in the adipose tissue [24,25]. Additionally, n-3 PUFAs reduce liver fat in patients and rodents with NAFLD [26,27].

Both n-3 PUFAs and vit D are among the most consumed supplements in the US for health promotion. Further, vit D deficiency, along with lower intake and low circulating n-3 PUFA levels, were observed in people with obesity [28]. Moreover, several studies have shown that n-3 PUFAs regulate vit D levels [29,30,31]. Clinical trials such as The VitaL (Vitamin D and Omega-3 Trial), which was the largest randomized placebo study, did not find evidence for lower incidence of cancer or cardiovascular disease with use of these supplements compared to placebo. However, these studies were conducted in older populations [32]. Further, subsequent research suggested that individuals with higher BMI displayed reduced responsiveness to vit D supplementation [33]. Whether a combination of vit D and n-3 PUFAs is beneficial in preventing obesity is unknown. Hence, we hypothesized that a combination of vit D and n-3 PUFAs will reduce diet-induced obesity in male C57BL6 mice through lipid lowering and anti-inflammatory actions in both adipose tissue and liver.

## 2. Materials and Methods

### 2.1. Mice and Diets

**Figure 1:**
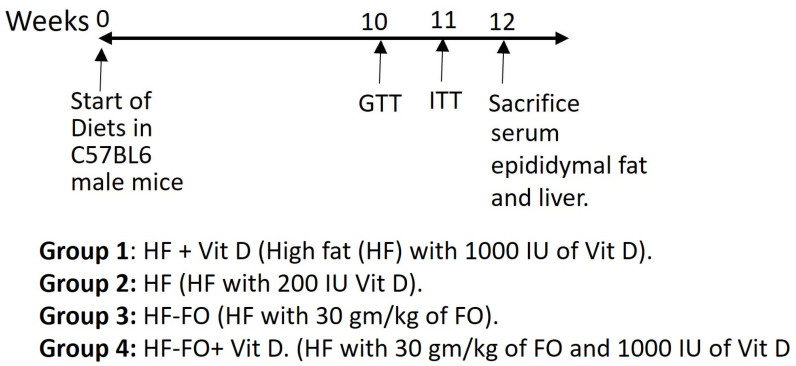
Study design and tests conducted in C57BL6J mice. Mice started the diets at 5 weeks of age.

All animal protocols were submitted to and approved by the Institutional Animal Care and Use Committee of Texas Tech University, protocol number 16001-04. Sixty male C57BL6 mice aged 4–5 weeks old were purchased from Jackson Laboratory (Bar Harbor, ME, USA). After one-week acclimatization, mice were split into four groups. Male mice (n = 15) were fed either a high fat (HF) diet supplemented with vitamin D (HF + vit D; 20, 32, 48% of energy from protein, carbohydrates, and 1000 IU of vit D), or an HF diet with a lower amount of 200 IU vit D (HF). The third group was supplemented with 30 gm/kg of FO (HF-FO) and 200 IU of vit D, and the last group was supplemented with 30 gm/kg of FO and 1000 IU of vit D (HF-FO + vit D), as shown in Figure 1. Menhaden oil, containing 35% of total omega-3 fatty acids, had a balanced concentration of EPA and DHA (a kind gift from Omega Proteins (Houston, TX, USA)). The mice were supplemented for 12 weeks with custom diets from Research Diets Inc. (New Brunswick, NJ, USA) and vitamin D from Sigma-Aldrich (St Louis, MO, USA). Detailed diet information is available in Supplemental Table S1. Casein stripped of vitamin D was used as a protein source. Micronutrients were maintained at the same level across all diet groups. Vitamin E was added to prevent lipid peroxidation. Male mice were individually housed in ventilated cages with ad libitum access to food and water. Animals were maintained on a 12-h day/night cycle at 23 °C. Mice were weighed weekly and their food was changed every week. Phenotypic tests, including glucose and insulin tolerance tests, were performed during the 10th and 11th weeks of the study. Body fat composition was measured using an Echo-MRI 3 in 1 analyzer (EchoMRI LLC, Houston, TX, USA) at 11 weeks of dietary intervention. At the end of the study, mice were fasted for 5 h and euthanized using CO_2_. Epididymal fat pad and liver were harvested and snap-frozen in liquid nitrogen for further analyses. Blood was collected using microtainer tubes (Thermo Fisher Scientific, Waltham, MA, USA) and centrifuged to obtain serum.

### 2.2. Glucose Tolerance Test (GTT) and Insulin Tolerance Test (ITT)

All groups of mice were fasted for 5 h for both GTT and ITT. Blood glucose was measured using a glucometer (Abbott Laboratories, Alameda, CA, USA). After measuring baseline glucose, all mice were injected intraperitoneally with 2 gm glucose/kg of body weight. Following injection, glucose was measured at 30, 60, 90, and 120 min intervals. For ITT, all mice were injected with 1 IU/kg body weight insulin (Humulin; Abbott, Chicago, IL, USA) intraperitoneally and blood glucose was measured at 15, 30, 45, 60, and 90 min intervals. Area under the curve (AUC) of GTT was assessed and calculated according to the trapezoidal method, as we have previously reported [34].

### 2.3. Immunohistochemical Staining

Epididymal fat pad and liver were fixed in Z-fix (Anatech Ltd., Battlecreek, MI, USA) overnight and transferred to 70% alcohol. The tissues were then paraffin embedded and stained with hematoxylin-eosin (H&E) (Thermo Fisher, Carlsbad, CA, USA). Images were captured using an EVOS^®^ FL Auto imaging system (Thermo Fisher Scientific, Waltham, MA, USA) at 20× magnification for epididymal fat and 40× magnification for the liver. Quantification of the adipocyte area was calculated for a minimum of 100 cells using a recently reported AdipoGauge software, version 1 for cellularity analyses [35].

### 2.4. Gene Expression

RNA was extracted using an isolation kit (Zymo Research, Irvine, CA, USA). RNA concentration and its purity were checked using Nanodrop (Thermo Fisher, Carlsbad, CA, USA). Total RNA was then reverse transcribed into cDNA using Maxima reverse transcriptase (Thermo Fisher, Carlsbad, CA, USA), followed by gene expression according to real-time quantitative polymerase chain reaction (RT-qPCR) using Sybr green master mix (Thermo Fisher Scientific, Waltham, MA, USA). qPCR was carried out using Quantstudio 3 (Thermo Fisher, CA, USA). The primers used in gene expression analyses were purchased from Sigma-Aldrich, St. Louis, MO, USA. The expression of individual genes was normalized to housekeeping gene 18s. Sequences of primers are provided in Supplemental Table S2. The qPCR results were calculated in the QuantStudio^TM^ (Version 5) software provided by Applied Biosystems (Thermo Fisher, CA, USA). Relative gene expression of target genes was normalized using the HF group as control and 18 S rRNA as housekeeping gene and the relative normalized expression was calculated using the delta delta comparative threshold method (ΔΔCt).

### 2.5. Serum Triglycerides

Serum triglycerides were measured using a colorimetric assay kit, 10010303 (Cayman Chemical, Ann Arbor, MI, USA) as per the manufacturer’s instructions. A total of 50 µL of serum was diluted 1:2 with standard diluent prior to the assay.

### 2.6. Statistical Analyses

Diet-specific differences were examined by one-way analysis of variance (ANOVA), followed by Tukey’s post-hoc test in GraphPad Prism (version 8). Results are presented as mean ± SEM (standard error of means), with statistical significance considered at *p* < 0.05.

## 3. Results

**Figure 2:**
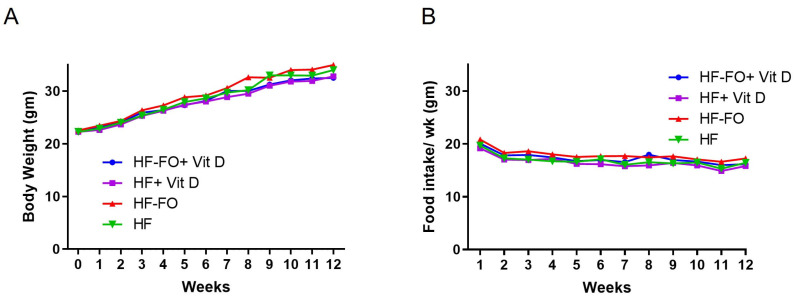
Fish oil and vit D effects on body weight in male mice: (**A**) weekly body weight in male mice and their (**B**) food intake. Data are presented as mean ± SEM. n = 15 for all groups.

Mice were randomly assigned to the four dietary groups and fed their respective diets for 12 weeks. We observed comparable body weight and food intake between the four dietary groups (Figure 2A,B). As other studies have shown significant metabolic changes without alterations in body weight with FO supplementation [24], we performed additional metabolic analyses.

**Figure 3:**
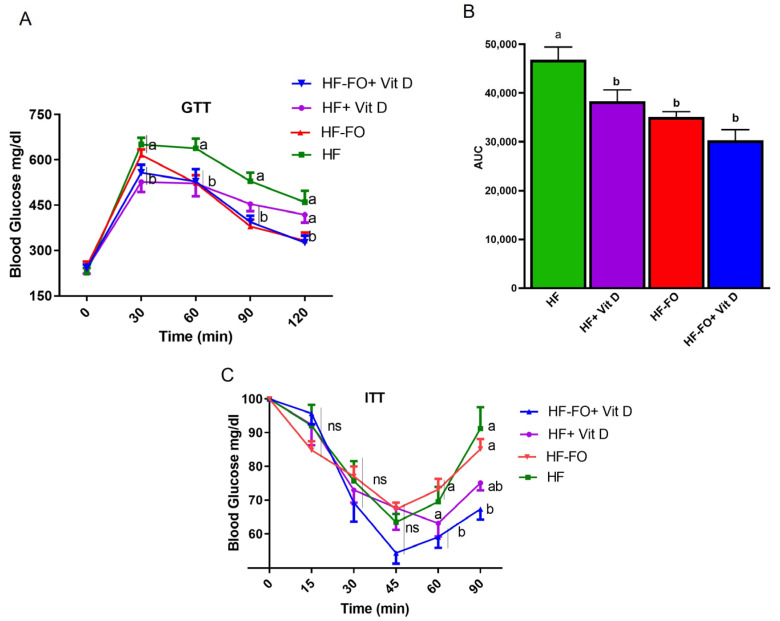
Fish oil and vit D effects on glucose clearance and insulin sensitivity in male mice: (**A**) glucose tolerance test (GTT) in male mice; (**B**) area under the curve for GTT; (**C**) insulin tolerance test (ITT) in male mice. Data are presented as mean ± SEM. n = 15 for all groups. Error bars with different letters are significantly different. (e.g., “a” is significantly different from “b”). *p* < 0.05 unless mentioned. For Figure 3B, HF vs. HF + vit D (*p* < 0.001), HF vs. HF-FO (*p* < 0.001), HF vs. HF-FO + vit D (*p* < 0.0001).

Interestingly, mice fed HF supplemented with vit D (HF + vit D) cleared glucose significantly (*p* < 0.05) rapidly, with lower blood glucose values at 60 and 90 min, compared to mice fed HF only (Figure 3A). Further, as shown by area under the curve (AUC), all groups had significantly (*p* < 0.05) better glucose clearance compared to mice fed HF, as shown in Figure 3B. For ITT, glucose levels of mice fed HF dropped to 65 mg/dl after insulin injection, followed by increase at 90 min, indicating insulin resistance (Figure 3C). Mice fed FO with no vit D supplementation (HF-FO) had similar insulin intolerance as mice fed HF. However, mice fed HF containing both FO and vit D (HF-FO + vit D) exhibited a significantly better response (*p* < 0.05) to insulin, as indicated by the lower blood glucose values at 60 and 90 min compared to HF and HF-FO groups (Figure 3C).

**Figure 4:**
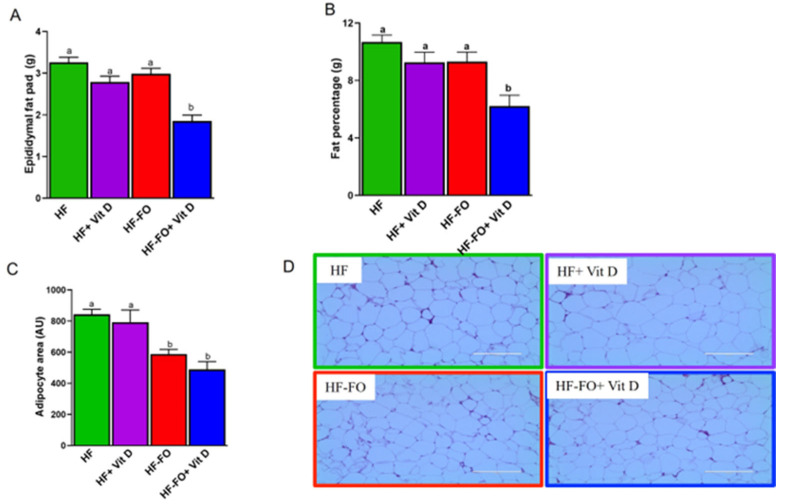
Fish oil combined with vit D supplementation altered overall adiposity in male mice: (**A**) epididymal fat pad weight; (**B**) body fat composition; (**C**,**D**) mean adipocyte cell area in male mice along with their representative images. Scale bar = 200 µM. Data are presented as mean ± SEM. n = 15 for Figure 4A–C and n = 4 for histology images. Error bars with different letters are significantly different. *p* < 0.05 unless mentioned. For fat percentage (Figure 4B), HF vs. HF-FO + vit D (*p* < 0.001).

We were specifically interested in understanding the role of FO and vit D in reducing adiposity and associated changes in cellularity. Hence, we measured both body fat content and epididymal fat pad weight at terminal sacrifice. Mice fed FO with vit D (HF-FO + vit D) had lower fat pad weights compared to all other groups (Figure 4A). Similar results were observed for body fat percentage, which was also significantly lower (*p* < 0.001) in the group fed vit D and FO (HF-FO + vit D), as shown in Figure 4B. We further determined whether these differences were due to changes in adipocyte size within the epididymal fat pad using hematoxylin-and-eosin-stained sections. Mice fed FO with or without vit D had significantly (*p* < 0.05) lower adipocyte area compared to mice fed HF, consistent with the fat pad and fat content data (Figure 4C,D).

**Figure 5:**
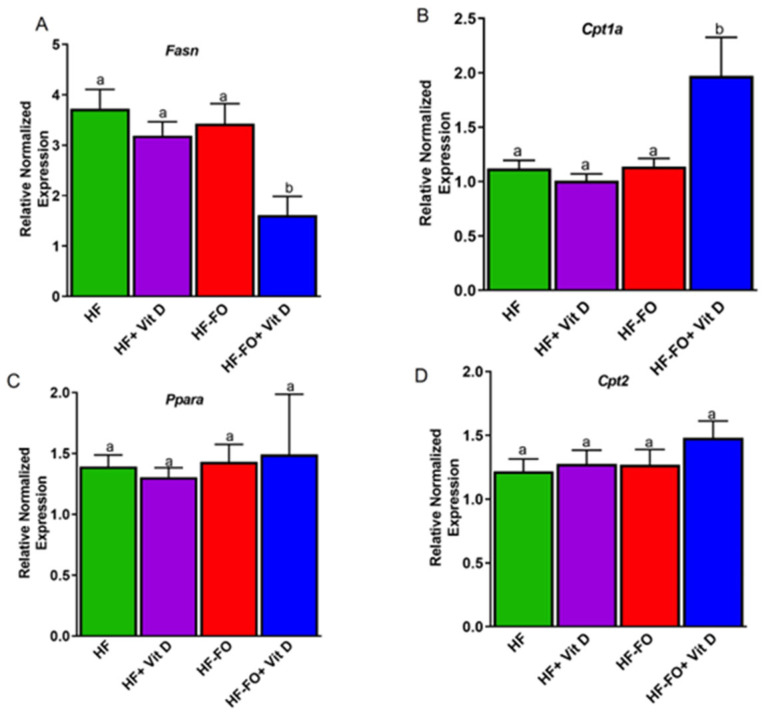
Fish oil and vit D combination altered mRNA levels of lipid metabolism markers in epididymal fat pad. mRNA levels of (**A**) fatty acid synthase (Fasn) in epididymal fat pad. (**B**–**D**) mRNA levels of fatty acid oxidation markers carnityl palmitoyl transferase-1 (cpt-1), peroxisome proliferator activated receptor alpha (Ppara), and carnityl palmitoyl transferase-2 (Cpt2) in epididymal fat pad of male mice. Data are presented as mean ± SEM. n = 12 for all groups. Error bars with different letters are significantly different. *p* < 0.05 unless mentioned. For Figure 5B, HF-FO + vit D vs. all other groups *p* < 0.001.

To better understand metabolic changes in adipose tissue accounting for the above differences in adiposity and cell size, we measured mRNA levels of lipid metabolism markers. Fatty acid synthase (*Fasn*) mRNA levels were significantly (*p* < 0.05) lower in the mice fed FO and vit D (HF-FO+ Vit D) compared to all other groups, suggesting that de novo lipogenesis was reduced with combined vit D and FO (Figure 5A). Further, genetic analyses of fatty acid oxidation were measured using markers such as carnitine palmitoyl transferase-1a (*cpt-1a*), *cpt2*, and peroxisome proliferator-activated receptor α (*Ppara*). In line with fatty acid synthesis, fatty acid oxidation measured by mRNA levels of *cpt-1a* was significantly (*p* < 0.001) higher in mice fed FO and vit D (HF-FO + vit D) (Figure 5B). However, no differences in mRNA levels of *Ppara* or *cpt2* were observed (Figure 5C,D).

**Figure 6:**
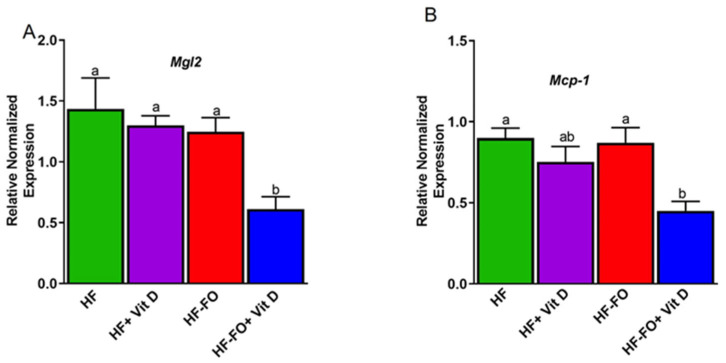
Fish oil and vit D supplementation alters mRNA levels of pro-inflammatory markers in mice epididymal fat pads: (**A**) mRNA levels of pro-inflammatory marker macrophage galactose N-acetyl-galactosamine specific lectin 2 (Mgl2) in mice, (**B**) monocyte chemoattractant protein-1. Data are presented as mean ± SEM. n = 12 for all groups. Error bars with different letters are significantly different. *p* < 0.05 unless mentioned. For Mgl2, HF-FO + vit D vs. HF-FO (*p* < 0.001).

As chronic low-grade inflammation is an underlying feature of obesity, we determined changes in mRNA levels of pro-inflammatory markers in adipose tissue, specifically macrophage markers. Macrophage galactose N acetyl galactosamine (*Mgl-2*) mRNA levels were significantly (*p* < 0.001) lower only in vit D and FO (HF-FO + vit D) groups compared to others (Figure 6A). For another inflammatory marker, monocyte chemoattractant protein-1 (*mcp-1*), mRNA levels were significantly (*p* < 0.05) lower in vit D and FO (HF-FO + vit D) groups compared to HF and HF+FO, but were comparable to the HF-D group (Figure 6B).

**Figure 7:**
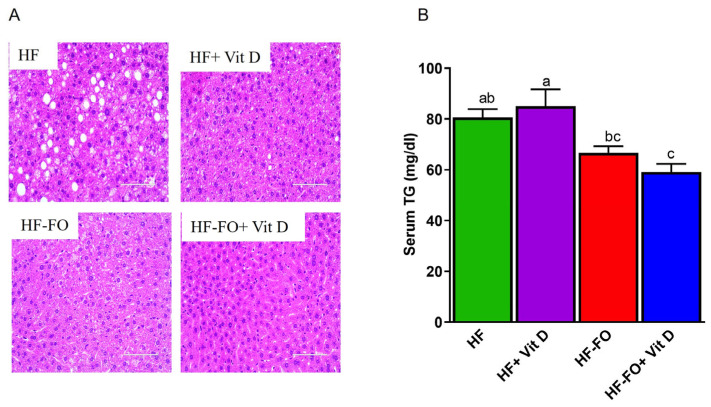
Fish oil and vit D effects in liver: (**A**) hematoxylin and eosin (H&E) staining of livers from male mice; (**B**) serum triglycerides measured by calorimetry. n = 12 for all groups for serum triglycerides and n = 4 for histology. Scale bar = 200 µM. Data are presented as mean ± SEM. Error bars with different letters are significantly different. *p*< 0.05 unless mentioned. For Figure 7B, HF-FO + vit D vs. HF + vit D (*p* < 0.001).

Excess adiposity leads to ectopic fat accumulation in other tissues including the liver, contributing to NAFLD. Hence, we investigated changes in lipid deposition in the liver under these dietary interventions. Only the HF group had higher fat deposits, as shown in Figure 7A, compared to the other groups. Groups supplemented with FO, vit D, or the combination had reduced fat deposits compared to the HF group. Further, serum triglycerides levels were significantly (*p* < 0.05) lower in both groups supplemented with FO, as shown in Figure 7B.

**Figure 8:**
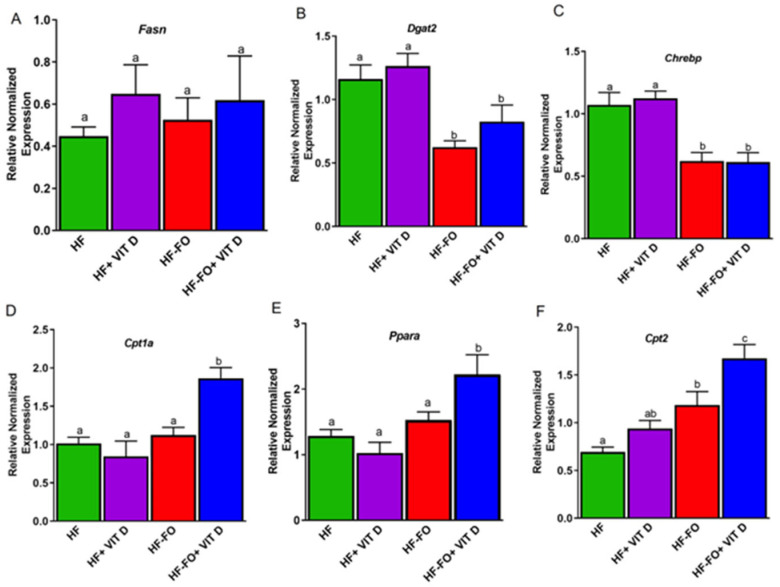
Fish oil and vit D altered markers of lipid metabolism in liver: (**A**) mRNA levels of fatty acid synthase (*Fasn*) in liver, (**B**) diacylglycerol O-acyltransferase-2 (*Dgat-2*), and (**C**) carbohydrate responsive element binding protein (*Chrebp*). (**D**–**F**) mRNA levels of fatty acid oxidation markers carnitine palmitoyl transferase-1 (*Cpt-1*), peroxisome proliferator activated receptor alpha (*Ppara*), and carnitine palmitoyl ferase-2 (*Cpt2*) in liver of male mice. n = 12 for all groups. Error bars with different letters are significantly different. *p* < 0.05 unless mentioned. For *Chrebp*, HF–vit D vs. HF-FO and HF-FO + vit D (*p* < 0.001). For Cpt1a, HF + vit D vs. HF-FO + vit D (*p* < 0.001). For Cpt2, HF vs. HF-FO + vit D (*p* < 0.0001) and HF–vit D vs. HF-FO + vit D (*p* < 0.001).

Similarly to adipose tissue, we measured mRNA levels of fatty acid synthesis markers in the liver but observed no differences in *Fasn* between the groups (Figure 8A). However, the mRNA levels of diacylglycerol O-acyltransferase-2 (*Dgat2*) responsible for the triglyceride synthesis were significantly (*p* < 0.05) lower in the FO groups compared to the groups supplemented with HF (Figure 8B). In line with Dgat2, mRNA levels of the carbohydrate response element gene (*Chrebp)* were significantly (*p* < 0.001) lower in the groups fed FO compared to the groups fed HF (Figure 8C). For fatty acid oxidation markers, *Cpt1a* and *Ppara*, mRNA levels were significantly (*p* < 0.05) higher in the group fed HF with FO and vitamin D (HF-FO + vit D) compared to other groups, as shown in Figure 8D,E. For *Cpt2*, mice fed HF with or without vit D had similar mRNA levels in the liver. However, the group supplemented with only FO (HF-FO) had significantly (*p* < 0.05) higher *Cpt2* mRNA levels compared to the HF group. Further, mice supplemented with FO and vit D (HF-FO + vit D) had significantly (*p* < 0.05) higher levels compared to other groups (Figure 8F).

**Figure 9:**
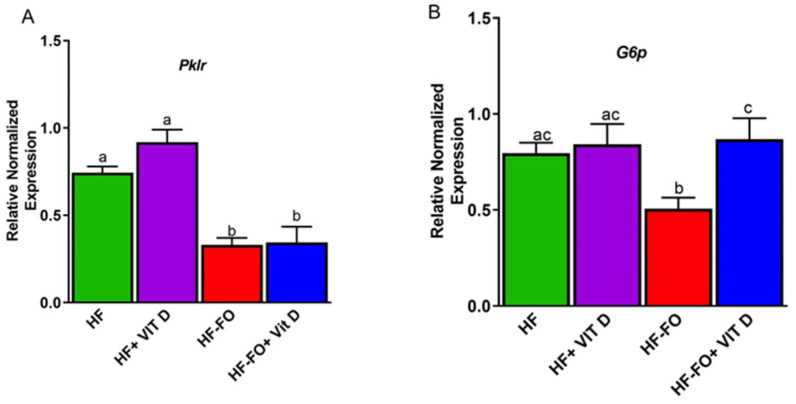
Fish oil and vit D alter markers of glycolysis in male mouse liver: (**A**) mRNA levels of pyruvate kinase (*pklr*), (**B**) glucose 6-phosphate (*G6P*). n = 12 for all groups. Error bars with different letters are significantly different. *p* < 0.05 unless mentioned. For Pklr, HF + vit D vs. HF-FO (*p* < 0.0001) and HF + vit D vs. HF-FO + vit D (*p* < 0.0001).

To measure glycolytic effects, we examined a gene involved in glycolysis, pyruvate kinase (*Pklr*), and mRNA expression of the gluconeogenic marker glucose 6 phosphatase (*G6p*). *Pklr* mRNA levels were comparable between groups fed HF and HF + vit D (Figure 9A). However, *Pklr* mRNA levels were significantly (*p* < 0.0001) lower in both groups supplemented with FO when compared to HF only (Figure 8A). *G6P* mRNA levels were comparable in the HF groups and in the mice supplemented with both vit D and FO; however, *G6P* gene expression was significantly (*p* < 0.05) lower in the mice fed HF with FO (HF-FO) (Figure 9B) compared to other groups.

## 4. Discussion

In the present study, we report the metabolic benefits of combined vit D and FO, which reduce adiposity and inflammatory markers in adipose tissue and lipid deposition in the liver of diet-induced obese male mice. We did not observe prominent effects when the mice were supplemented with either FO or vit D alone, as compared to the combination of these two bioactive dietary compounds. We further investigated the molecular mechanisms mediating the effects of vit D and FO. When FO was supplemented with vit D, markers of fatty acid oxidation were increased in the liver, whereas only one marker of fatty acid oxidation was altered in the adipose tissue. Further, we did not observe an additive effect of FO and vit D. It seems probable that FO showed potential beneficial effects in the presence of vitamin D and some of the beneficial effects were therefore absent with insufficient amounts of vit D.

Our mice supplemented with vit D and FO had comparable body weight to those supplemented with HF or only vit D. This is consistent with other rodent and clinical studies reporting that vit D supplementation did not alter the body weight [36,37,38,39]. In rodents, intraperitoneal injection of vit D reduced body weight compared to oral administration [40]. This route bypasses the liver, leading to more pronounced effects of vit D [41].

Interestingly, for GTT, all treatment groups (vit D, FO, and vit D + FO) had improved glucose clearance compared to HF as shown by AUC data, with no synergistic benefits of the combined supplementation compared to individual treatment of vit D or FO. FO is well documented for improving glucose clearance in mice [24,25,42]. Interestingly, ITT showed that insulin resistance was lower in the group with FO and vit D compared to the other groups, indicating that the combination improved insulin sensitivity, which may be due to their predominant activity in adipose tissue and muscle. Clinical studies with vit D supplementation did not find improvements in homeostatic model assessment for insulin resistance (HOMA IR), which is an accepted measure of insulin sensitivity. In line with our study, others reported that vit D, even up to 15000 IU, did not improve glucose homeostasis in mice [36].

Diet-induced obesity is associated with chronic low-grade inflammation and infiltration of macrophages into adipocytes. Hence, we measured markers of inflammation in adipose tissue. However, contrary to previous reports from our lab and others that reported anti-inflammatory effects of FO, the current study did not observe a reduction in inflammation with FO alone [27,42,43]. This discrepancy may be attributed to the fact that the group receiving only FO had low levels of vitamin D (200 IU), implying that a certain level of vitamin D may be necessary to detect the anti-inflammatory effects of FO. Further, in the current study, we used menhaden FO, which contains equal amounts of EPA and DHA. In contrast, our previous studies employed EPA-enriched FO (36 gm/kg) [24] or diets rich in EPA (98% pure ethyl ester of EPA), which produced more potent metabolic effects, thus possibly explaining the dampened effects of FO in this study compared to others using EPA or EPA-enriched oils. Interestingly, only in the presence of vit D supplementation does FO show adiposity-reducing effects. Studies have shown that vit D by itself reduces inflammation in adipose tissue, as well in mouse and human adipocytes [36,44]. Vit D was reported to reduce inflammation in part through the NFKB pathway, which is also the same pathway that mediates the anti-inflammatory functions of FO [44,45,46]. Consistent with our findings, clinical studies which used 2000 IU of vit D daily did not detect a decrease in C-reactive protein (CRP) levels [47]. However, studies that used a very high dose of 50,000 IU vit D weekly reported reduction in CRP. These differences could be due to differences in the duration and dosage of vit D.

Excessive adiposity contributes to ectopic fat build up in the liver, leading to hepatic steatosis and inflammation. Thus, we analyzed changes in lipid metabolism and inflammation in the liver. Further, vit D deficiency is known to lead to NAFLD [16,48,49,50]. With vit D supplementation alone, no changes in fatty acid synthesis were observed in liver. However, FO with or without vit D reduced *dgat2* and *Chrebp*, suggesting downregulation of triglyceride synthesis and glycolysis, respectively, in the liver. Notably, only the combination of vit D and FO improved fatty acid oxidation in the liver. Higher doses of vit D may be necessary for effects to manifest, potentially explaining the lack of effects in our study. Future extensive dose response studies with vit D are warranted.

We predicted that vit D would increase fatty acid oxidation and reduce inflammation in high-fat-fed mice. However, we did not observe such effects when vit D was supplemented alone. Probable reasons for this discrepancy could include higher adipose mass with HF supplementation. This could result in a smaller amount of vit D distributed within a large reservoir of fat mass, leading to dilution of vit D in the adipose tissue, and subsequently reducing its effectiveness [51]. Supporting this theory of the need for a reduced reservoir of vit D, studies have also reported increased vit D levels after bariatric surgery [52]. This suggests that individuals with obesity may need a higher dose of vit D compared to lean individuals to see similar effects, owing to the dilution of vit D in adipose tissue. Similarly, in a normal healthy mouse, serum concentration of 25 hydroxy vit D reaches 120 ng/mL when supplemented with 1000 IU vit D/kg of diet [53]. However, in a diet-induced obese mouse model, serum concentration reached only 7.14 ng/ml even with 15,000 IU of vit D supplementation, likely due to volumetric dilution of vit D caused by larger adipose tissue mass [54]. Our dosage was significantly lower than those in studies elucidating the mechanism in adipose tissue, suggesting that, in our study, vitamin D may not have reached sufficient levels to exert its impact [41]. This underscores the necessity for future studies to evaluate higher concentrations of vitamin D than currently recommended, specifically in subjects with obesity.

One of the limitations of this study is that we used a higher dose of FO. We used 30 gm/kg of menhaden FO, which had balanced levels of both EPA and DHA, to match amounts used in our previous studies [55,56]. This translates to over 10 g FO per day in humans. Even though it is higher, clinical studies have used higher doses with no toxic or serious side effects [57]. Another limitation is not having a low-fat group that is also supplemented with FO and/or vit D to determine the metabolic benefits of these dietary components for lean subjects. Moreover, we studied the impact of FO and vit D only in male C57BL6 mice, given their susceptibility to diet-induced obesity [58]. However, it is necessary to investigate these effects in female mice in future, despite their resistance to diet-induced obesity.

In conclusion, our data provide evidence that FO and vit D in combination reduced markers of obesity in adipose tissue and the liver. However, more mechanistic experiments are required using dose response studies that incorporate low and higher doses of vit D (we have done such dose studies with FO) [24]. Our findings therefore suggest that vit D deficiency may prevent the protective metabolic effects of FO. Hence, supplementation of vit D along with FO could be beneficial in treating metabolic diseases, including obesity, insulin resistance, and NAFLD.

## Data Availability

Data would be provided by the author on request.

## Supplementary Materials

The following supporting information can be downloaded at: https://www.mdpi.com/article/10.3390/biom14040474/s1, Table S1: Dietary information; Table S2: Fatty acid composition of menhaden oil.
