# Supplementary material for: Enhanced Metabolic Effects of Fish Oil When Combined with Vitamin D in Diet-Induced Obese Male Mice
Source: Biomolecules. 2024 Apr 12;14(4):474. doi: 10.3390/biom14040474 (PMC11048485; doi:10.3390/biom14040474)
Supplement: Supplementary file 1 [file biomolecules-14-00474-s001.zip › biomolecules-2919902-supplementary.pdf]

Table S1. Diet composition.

	**High Fat-Fish oil**		**High Fat**		**High Fat + Vit D**		**High Fat-Fish oil + vit D**	
	g	*kcal%*	g	*kcal%*	g	*kcal%*	g	*kcal%*
Protein	24.2	*20*	24.2	*20*	24.2	*20*	24.2	*20*
Carbohydrate	38.2	*32*	38.2	*32*	38.2	*32*	38.2	*32*
Fat	26	*48*	26	*48*	26	*48*	26	*48*
Total		*100*		*100*		*100*		*100*
kcal/g	4.84		4.84		4.84		4.84	
**Ingredient**	**g**	***kcal***	**g**	***kcal***	**g**	***kcal***	**g**	***kcal***
Casein, 30 mesh	200	*800*	200	*800*	200	*800*	200	*800*
L-Cystine	3	*12*	3	*12*	3	*12*	3	*12*
Corn Starch	38	*152*	38	*152*	38	*152*	38	*152*
Maltodextrin 10	100	*400*	100	*400*	100	*400*	100	*400*
Sucrose	173	*691*	173	*691*	173	*691*	173	*691*
Cellulose	50	*0*	50	*0*	50	*0*	50	*0*
Soybean Oil	25	*225*	25	*225*	25	*225*	25	*225*
Lard	144	*1296*	193	*1737*	144	*1296*	193	*1737*
Vitamin D IU	200	*0*	200	*0*	1000	*0*	1000	*0*
Menhaden Oil, Nutegrity	49	*441*	0	*0*	49	*441*	0	*0*
Mineral Mix, S10026	10	*0*	10	*0*	10	*0*	10	*0*
DiCalcium Phosphate	13	*0*	13	*0*	13	*0*	13	*0*
Calcium Carbonate	5.5	*0*	5.5	*0*	5.5	*0*	5.5	*0*
Potassium Citrate, 1 H2O	16.5	*0*	16.5	*0*	16.5	*0*	16.5	*0*
Vitamin Mix, V10001	10	*40*	10	*40*	10	*40*	10	*40*

Choline Bitartrate	2	*0*	2	*0*	2	*0*	2	*0*
Vitamin E Acetate, 50% (500 IU/g)	0.13	*0*	0.13	*0*	0.13	*0*	0.13	*0*
**Total**	**839**	***4057***	**839**	***4057***	**839**	***4057***	**839**	***4057***

Table S2. Primer Information

Primer Name	Forward	Reverse
Fasn	TGTATCCTGCTGTCCAACCT	GGCTTGTCCTGCTCTAACTG
Ppara	ATCCACGAAGCCTACCTGAA	AATCGGACCTCTGCCTCTT
Cpt1a	GAGACAGACACCATCCAACAC	GAGCCAGACCTTGAAGTAACG
Cpt2	CAGCACAGCATCGTACCCA	TCCCAATGCCGTTCTCAAAAT
Mcp-1	ACTTCTATGCCTCCTGCTCAT	CCTGCTTGTGATTCTCCTGTAG
DGat2	CCTCATCGCCGCCTACTC	GAGCCAGGTGACAGAGAAGAT
Chrebp	GACTCGGACTCGGATACG	GCTGTGGATGACCTGTGA
Pklr	TTCCTCCGAGTTGGTGAT	TGGCTAGATGGCAGATGT
G6p	AACGCCTTCTATGTCCTCTTTC	GTTGCTGTAGTAGTCGGTGTCC
18S	GTTGCTGTAGTAGTCGGTGTCC	TGCCAGAGTCTCGTTCGTTA
